# Effectiveness of the forced‐choice coin test for detecting malingering during forensic psychiatric examinations

**DOI:** 10.1002/pcn5.87

**Published:** 2023-04-13

**Authors:** Keisuke Tsuji

**Affiliations:** ^1^ Faculty of Human Sciences Musashino University Tokyo Japan

**Keywords:** forensic psychiatric examination, forensic psychiatry, intellectual disability, malingering, psychometrics

## Abstract

**Background:**

In general clinical psychiatric practice, open questions are favored over closed ones because they are considered more therapeutically effective and less likely to make the patients pander to us. However, in forensic psychiatric examinations, suspects may attempt malingering.

**Case Presentation:**

Using a simple examination based on a forced‐choice technique, the author proved that the level of intelligence of a theft suspect pretending to have an intellectual developmental disorder was not so low. The author prepared two sets consisting of a few coins each and asked the suspect to choose which set had a higher total value. The suspect was questioned repeatedly over multiple trials. He always selected the wrong set over the course of more than 10 trials.

**Conclusion:**

If the suspect really did not know the correct answer, the probability of getting the answer right or wrong in a binary choice question is 50% for both. The probability of answering the question wrong by chance 10 times in a row is (1/2),^10^ in other words, about 0.1%. It was evident that the suspect intentionally kept answering incorrectly. When suspects who pretend to have an intellectual developmental disorder answer only “I do not know” to all questions without actively playing out the symptoms, it is difficult to demonstrate that the symptoms are psychiatrically conflicting and prove that they are malingering. Even in such cases, this type of test based on a forced‐choice technique can be used to prove that suspects are behaving falsely.

## Graphical abstract

In forensic psychiatric examinations, suspects or defendants may attempt malingering. Even in such cases, the forced‐choice coin test based on a forced‐choice technique can be used to prove that suspects or defendants are behaving falsely.

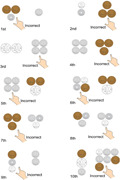

## BACKGROUND

In general clinical psychiatric practice, doubting every word of a patient makes treatment more difficult and hinders the development of a trusting rapport with patients, therefore many psychiatrists practice under the assumption that their patients are telling the truth. Even if a patient malingers, most such patients face difficulties and conflicts, and the goal of clinical psychiatry is to resolve such difficulties and conflicts. Simply exposing malingering will not heal the patients or help them adapt to society. Worse yet, it may lead to the development of new symptoms. At times, a clinician may have to put the pursuit of the truth on hold. For instance, it is normal for patients with hysteria to exaggerate their symptoms. Malingering is rarely recorded in medical records.

However, forensic psychiatric examinations must consider the possibility that suspects or defendants may exaggerate symptoms or act out nonexistent symptoms as if they exist to avoid punishment for a crime. Even operational diagnostic criteria, such as the Diagnostic and Statistical Manual of Mental Disorders and the International Classification of Diseases, suggest that malingering must be kept in mind during examinations in forensic psychiatric medicine, and state that malingering is a differential diagnosis that must always be considered.^1^, ^2^, ^3^, ^4^

Rather than closed questions, which force the patients to select an answer from a choice of options, open questions allow patients to express themselves freely, lowering the risk of making them pander to us and influencing their answers. Open questions are also considered to be therapeutically effective. Not only in general clinical practice, but also in forensic psychiatric examinations, the latter type of questioning is the basis of interviews. On the other hand, in the case of suspects or defendants who malinger or make false statements, we need to ask questions that let them choose a clear answer without ambiguity—in other words, a forced‐choice technique—and then let them tell a clearly false lie so that we can prove that they are speaking falsely.^5^ Forced‐choice techniques have various application possibilities.^6^, ^7^, ^8^, ^9^ The author of this study proved that the perpetrator was malingering by using a forced‐choice technique‐based test as a forensic examination for a case where a thief was pretending to have an intellectual developmental disorder.

## CASE PRESENTATION

**A** (alleged male theft, 41 years of age at the time of the offense)

**A** had no significant developmental abnormalities and attended regular classes in elementary and junior high school, although he performed poorly academically. **A**'s father died of illness when **A** was in elementary school, and thereafter **A** lived in a juvenile independence support facility. After graduating from junior high school, **A** worked at various places such as a janitorial company, a dry cleaner, a family restaurant, a Japanese‐style inn (*ryokan*), and a small factory, but he did not last long in any of these jobs. At age 15 years, he obtained an intellectual developmental disorder ID booklet. The classification level in his intellectual developmental disorder ID booklet was level 4, equivalent to mild intellectual developmental disorder. At age 18 years, **A** left the juvenile independence support facility and moved around from place to place, which included working for a short time as a live‐in a Japanese‐style inn with hot spring baths (*onsen ryokan*). Although he became homeless at one point, he also had fun being a fanatical fan of a Japanese idol girl group, watching baseball games, and frequenting red‐light districts. At age 40 years, he was arrested for theft from inside the changing rooms of public baths and hot springs, and sentenced to 1 year in prison with 3 years of probation.

At age 41 years, he falsified his personal and family backgrounds and obtained a job as a waiter and janitor at an *onsen ryokan*, working as live‐in staff while residing in the staff dormitory. A few days after getting the job, he stole a few million yen in cash he found stored in a meeting room inside the staff dormitory. Pretending he was going to return soon, he packed all of his belongings and quietly fled the *onsen ryokan*. When the owner of the *onsen ryokan* noticed that the cash had gone missing, the owner became suspicious of **A**, who had disappeared, and called **A** on his cell phone, but **A** did not answer. **A** deposited the stolen money into his own bank account and blew it all buying merchandise from a Japanese idol girl group, dating call girls from escort services, buying lavish gifts for the call girls, and traveling to many places with them. After **A** was arrested, he admitted to stealing the cash for personal entertainment. Although he expressed remorse, he denied having planned the crime.

As the suspect had an intellectual developmental disorder ID booklet, a forensic psychiatric examination was conducted by the author to determine whether the suspect could be tried under the normal judicial system. During the interview, **A** had a poor posture and a dull facial expression. The suspect understood the intentions of the questions. He exhibited no psychotic symptoms, no disturbances of consciousness or thinking, and no fluctuations of mood or motivation. Nevertheless, no matter what he was asked, he answered, “I‐do‐not‐know” in an overly slow manner, carefully pronouncing every single syllable. When he was given single‐digit addition and subtraction problems, he replied, “I‐do‐not‐know.” He refused to take the Baum test, and his Mini‐Mental State Examination score was 3 points. Even before the questions were asked, he insisted that “Books I only look, I do not understand. Even if *hiragana* (Japanese phonetic characters that are simpler and easier to read than Chinese characters) are there, I do not understand well. Newspaper I only look, I do not understand.” He was clearly acting suspiciously. For instance, he started to speak faster as the conversation continued, but realizing that his speech was gaining speed, he went back to speaking slowly. He also suddenly became silent when the topic shifted to the case.

Because of his poor academic performance, **A** was educated only up to the end of junior high school. However, despite his poor social and vocational adaptation and being unskilled, he could still obtain several jobs, and though imperfect, he had a social life. His lavish spending spree until being arrested after fleeing with a large amount of stolen cash was indicative of him having a certain level of intellectual capacity. At first glance, he did not seem clever; however, his demeanor during the interview indicated that he had a fair level of comprehension, thinking, and decision‐making skills. Although the classification levels of the intellectual developmental disorder ID booklet may sometimes be assessed slightly more severely to qualify for more attentive services, one is rarely classified into a less severe level. There were no signs that the suspect suffered from an acquired mental illness resulting in declined intelligence.

Pretending to be highly intelligent is difficult, but it is relatively easy to pretend to be less intelligent. **A**'s intellectual ability was considered to be between borderline intellectual functioning and mild intellectual development disorder, but it was clear that he was trying to appear less intelligent than he really was. It was clear that **A** was malingering, as demonstrated by (i) his uncooperativeness toward medical examinations and tests, (ii) the disparities between his claims and objective findings, (iii) his constantly changing stories when contradictions were pointed out, and (iv) his suspicious behavior. When suspects or defendants pretend to have an intellectual developmental disorder or a major neurocognitive disorder, it might look suspicious to an experienced psychiatrist. However, it is quite difficult to prove rationally that the suspect or defendant is malingering. Nevertheless, conclusive evidence was needed to prove in court that **A** was pretending to be of low intelligence.

The author prepared two sets consisting of a few coins each and repeatedly asked **A** to choose the set with a higher total value. This convenient method can be administered to examinees who are not very cooperative in taking tests. In this study, this test based on the forced‐choice technique is referred to as the forced‐choice coin test.

**Figure 1:**
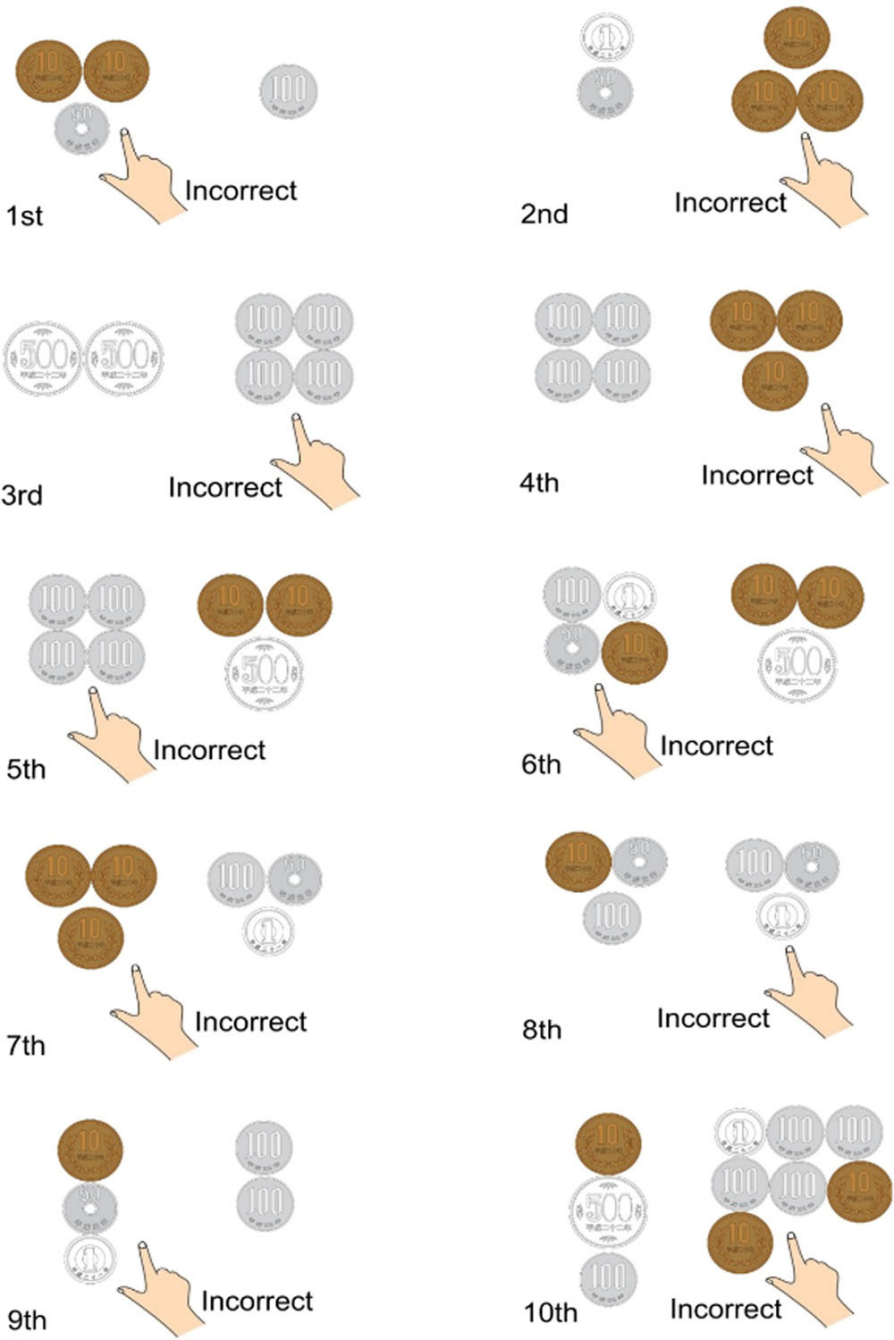
The author prepared two sets consisting of a few coins each and repeatedly asked **A** to choose the set with a higher total value. **A** always got the answers wrong.

In the first trial, **A** was presented with two sets of coins: one set had a total of three coins, consisting of two 10‐yen coins and one 50‐yen coin; the other set had one 100‐yen coin. When **A** was asked which set has a higher total value, he pointed at the set that had the two 10‐yen coins and one 50‐yen coin. In the second trial, **A** was presented with a set that consisted of a total of two coins (one 1‐yen coin and one 50‐yen coin). The other set had three 10‐yen coins. When **A** was asked which set has a higher total value, he pointed at the set with three 10‐yen coins. In this manner, **A** was repeatedly asked which of the two sets of coins had a higher total value, and he repeatedly answered incorrectly. Such trials were repeated more than 10 times. When questioning the suspect, the coins were laid out so that sometimes the correct answer would be on the left side, and at other times the correct answer would be on the right. Other times, the higher total value had fewer coins, while at other times, the higher total value consisted of more coins. Yet, **A** always got the answers wrong (see Figure 1).

## DISCUSSION

Answering incorrectly at first glance may seem to be due to low intelligence. However, if he really did not know the correct answer, the probability of correctly answering a binary choice question would be about 50%. The probability of repeatedly answering incorrectly 10 times in a row is (1/2)^10^ = 1/1024 ≈ 0.001, which is about 0.1%. It is clear that **A** intentionally continued to choose the wrong answers. This test used a variety of different coins, from 1‐yen coins to 500‐yen coins, which meant that one needed to do three‐digit additions. In other words, **A** could do three‐digit additions, yet pretended not to be able to do so. Thus, it proved that **A** was lying and trying to appear less intelligent.

To present a compelling argument in court for this case, it was necessary to explain that the probability of this phenomenon occurring by chance was only about 0.1%, which explains why **A** was asked to do the coin test 10 times. If the intent was only to determine statistical significance at the 5% level or *P* < 0.05, performing the test five times would have been adequate because (1/2)^5^ = 1/32 = 0.03125 < 0.05. Even to determine significance at the 1% level or *P* < 0.01, seven times would have sufficed because (1/2)^7^ = 1/128 = 0.0078125 < 0.01. From a statistical perspective, showing significance at *P* < 0.05 would have been sufficient. However, if the emphasis is on appealing to the judge, jury members, and lay judges in the case of Japan, showing significance at *P* < 0.001 might be more persuasive. This may be an area where one could flexibly adapt to the circumstances.

If the coin test is conducted 10 times, as in this case, and the examinee gets nine incorrect answers and one correct answer, the *P* value would be *P* = (_10_C_0_ + _10_C_1_)/2^10^ = (1 + 10)/2^10^ = 11/1024 ≈ 0.0107. Although this fails to reach *P* < 0.01, it does achieve *P* < 0.05. Hence, we could say that the examinee purposely answered incorrectly. If the examinee gets eight incorrect and two correct answers, as one might expect, *P* < 0.05 will no longer be achieved as *P* = (_10_C_0_ + _10_C_1_ + _10_C_2_)/2^10^ = (1 + 10 + 45)/2^10^ = 56/1024 ≈ 0.0546. However, if the coin test is done 20 times instead of 10, and if the examinee gets up to four correct answers, *P* < 0.01 will be reached because *P* = (_20_C_0_ + _20_C_1_ + _20_C_2_ + _20_C_3_ + _20_C_4_)/2^20^ = 6196/1,048,576 ≈ 0.00590. (Incidentally, even with five correct answers, significance is achieved at *P* < 0.05.) Being able to discuss the forced‐choice coin test at the level of high school mathematics will lead to its ease of use.

A very cautious suspect or defendant who is more intelligent than **A** may produce a compelling mixture of correct and incorrect answers. Further research is needed to address the evaluation methods for such cases. However, when this same forced‐choice coin test was used in another case, the author observed that the suspect started sporadically mixing correct answers after giving wrong answers for a while. In the case of that suspect, however, the percentage of wrong answers was overwhelmingly high. When the test was done 22 times, the suspect had 17 incorrect and only five correct answers. If the suspect had answered randomly, the probability of only getting five or fewer correct answers out of 22 tries is *P* = (_22_C_0_ + _22_C_1_ + _22_C_2_ + _22_C_3_ + _22_C_4_ + _22_C_5_)/2^22^ = 0.00845. As it is only 1% or less, it is very unlikely that this happened by chance. If we use the forced‐choice coin test in many cases and continue accumulating findings, we might be able to establish effective evaluation methods for highly intelligent and very cautious suspects and defendants.

In the case presented in this study, the author spontaneously came up with the coin combinations in front of **A**. Perhaps deciding on the combination of coins beforehand will improve the reliability and validity of the forced‐choice coin test. When deciding which set of coins to assign a higher value, the difficulty level should be set where it is easy for people of normal intelligence. This is an issue to be addressed in the future.

Suspects or defendants who pretend to be of low intelligence tend to answer “I do not know” to all kinds of questions, but **A** did not refuse and responded to the questions. This is an advantage of closed over open questions. While some suspects or defendants in the past were slightly reluctant to answer, according to the author's past experiences, few continued to refuse to answer when they were nudged with phrases such as “Relax and just try” or “It is okay to be wrong. Just choose the one that you think is correct.”

**Figure 2:**
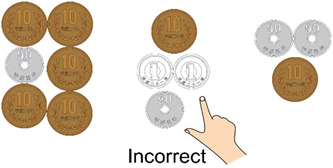
If three‐choice questions are adopted, it might be more effective to make three sets of coins where one of the sets is incorrect and the remaining two are correct.

It might be worth considering three‐choice questions with “I do not know” as one optional answer instead of binary choice questions where the examinee must choose between two sets of coin combinations. However, many examinees may answer “I do not know,” making it difficult to distinguish whether examinees are pretending not to know or actually do not know. If three‐choice questions are adopted, it might be more effective to make three sets of coins where one of the sets is incorrect and the remaining two are correct (see Figure 2). If a suspect or defendant continues to provide incorrect answers in this three‐choice question format, then three trials will be sufficient to reach *P* < 0.05 (*P* = (1/3)^3^ ≈ 0.0370), five to achieve *P* < 0.01 (*P* = (1/3)^5^ ≈ 0.00411), and seven to satisfy *P* < 0.001 (*P* = (1/3)^7^ ≈ 0.000457). It would be interesting to consider different methods, such as four‐ and five‐choice questions, in this manner. This is another issue to be addressed in the future.

The forced‐choice coin test used in this study is based on the forced‐choice technique that uses closed questions. Open questions are recommended in interrogations to prevent pandering to us. However, preventing pandering to us and spotting false statements are, in essence, at opposite ends of the spectrum. Furthermore, the forced‐choice coin test is a convenient test that can be administered by only pointing at the answer. It can be easily administered to examinees who are not very cooperative when undertaking medical examinations and tests. Although the forced‐choice coin test superficially asks, “Which has the higher total value?” the real aim is to ask, “Do you know which has the higher total value?”, thus the test is not affected by the examinee's lie. If anything, the goal is to get the examinee to lie.

When a malingerer actively acts out noticeable symptoms such as hallucinations and delusions, malingering can be proven if we can demonstrate that those symptoms differ from psychiatric findings, in other words, by showing that no disease presents such hallucinations and delusions.^10^, ^11^, ^12^, ^13^ However, when a person pretending to have a congenital or acquired intellectual disability, such as an intellectual development disorder or a major neurocognitive disorder, does not attempt to act out symptoms actively and answers only “I do not know” to all questions, it is difficult to detect malingering by simply pointing out the contradictions in the symptoms being acted out. The use of the forced‐choice technique for complaints of congenital or acquired intellectual disability is extremely rare.^14^ However, in forensic psychiatric examinations, the use of the forced‐choice technique, such as the forced‐choice coin test, should be considered in cases involving complaints of congenital or acquired intellectual disability.

Although “forced” is part of the term “forced‐choice technique,” it simply means that we are not looking for open‐ended answers. Thus, the examinee is not being forced to answer. This is why the author did not explain to **A** his right to remain silent. Unlike police and prosecutorial interrogations, there are no requirements for forensic psychiatric examinations in Japan to explain one's right to remain silent. However, as the method presented in this study aims to expose the inner thinking of suspects or defendants, in countries and regions where an individual's right to remain silent is valued, the test might need to be conducted after letting the individual know that they do not have to take the test if they do not want to.

## AUTHOR CONTRIBUTIONS

Keisuke Tsuji alone designed the study, the main conceptual ideas, and the proof outline, collected the data, interpreted the results, and wrote the manuscript.

## CONFLICT OF INTEREST STATEMENT

The author declares no conflict of interest.

## ETHICS APPROVAL STATEMENT

This study was conducted according to the principles of the Declaration of Helsinki.

## PATIENT CONSENT STATEMENT

Written informed consent for a forensic psychiatric examination was obtained from the suspect.

## CLINICAL TRIAL REGISTRATION

Not applicable.

## Data Availability

Not applicable.
